# Efficient Gearbox Fault Diagnosis Based on Improved Multi-Scale CNN with Lightweight Convolutional Attention

**DOI:** 10.3390/s25092636

**Published:** 2025-04-22

**Authors:** Bin Yuan, Yaoqi Li, Suifan Chen

**Affiliations:** College of Mechanical and Energy Engineering, Zhejiang University of Science and Technology, Hangzhou 310013, China; 13777680961@163.com (Y.L.); chensuifan8121@163.com (S.C.)

**Keywords:** dynamic convolutional kernel, EMD, gearbox fault diagnosis, lightweight convolutional attention, multi-scale CNN

## Abstract

As a core transmission component of modern industrial equipment, the operation status of the gearbox has a significant impact on the reliability and service life of major machinery. In this paper, we propose an intelligent diagnosis framework based on Empirical Mode Decomposition and multimodal feature co-optimization and innovatively construct a fault diagnosis model by fusing a multi-scale convolutional neural network and a lightweight convolutional attention model. The framework extracts the multi-band features of vibration signals through the improved multi-scale convolutional neural network, which significantly enhances adaptability to complex working conditions (variable rotational speed, strong noise); at the same time, the lightweight convolutional attention mechanism is used to replace the multi-attention of the traditional Transformer, which greatly reduces computational complexity while guaranteeing accuracy and realizes highly efficient, lightweight local–global feature modeling. The lightweight convolutional attention is adaptively captured by the dynamic convolutional kernel generation strategy to adaptively capture local features in the time domain, and combined with grouped convolution to enhance the computational efficiency further; in addition, parameterized revised linear units are introduced to retain fault-sensitive negative information, which enhances the model’s ability to detect weak faults. The experimental findings demonstrate that the proposed model achieves an accuracy greater than 98.9%, highlighting its exceptional diagnostic accuracy and robustness. Moreover, compared to other fault diagnosis methods, the model exhibits superior performance under complex working conditions.

## 1. Introduction

Traditional fault diagnosis methods primarily rely on vibration signal analysis, such as time-domain, frequency-domain, and time–frequency-domain techniques [1,2]. These methods perform well under steady-state conditions. However, their diagnostic capabilities may become limited when handling complex operating conditions such as variable speeds and strong noise interference, where the signals exhibit nonlinear and non-stationary characteristics. To address these limitations, deep learning techniques have gained significant traction in fault diagnosis, with architectures such as convolutional neural networks (CNNs) and long short-term memory (LSTM) being widely adopted. [3,4] They are commonly applied due to their ability to capture important features in fault signals. S. Zare et al. [5] employed a multi-channel convolutional neural network for fault diagnosis in mechanical equipment, achieving high accuracy. Notably, these intelligent methods also face data dependency challenges and may exhibit performance degradation when the training samples are insufficient. Recent research shows that multi-sensor data fusion technology can effectively enhance diagnostic robustness, particularly in methods based on flexible tensor singular value decomposition (SVD) [6]. Huang JF et al. further proposed flexible tensor SVD [7], overcoming the inherent limitations of the currently popular tensor singular value decomposition (tensor SVD) method based on n-mode products. By jointly analyzing multi-channel signals such as vibration and temperature, this approach significantly improves signal processing capabilities and diagnostic performance under complex working conditions. Under small-sample conditions, deep transfer learning strategies are also an effective way to improve fault diagnosis efficiency. The method proposed by Djaballah S et al. [8], which combines CWT time–frequency images with CNNs, not only reduces training time, but also enhances diagnostic accuracy. Jalayer M et al. [9] innovatively proposed convolutional long short-term memories (CLSTMs) combined with fast Fourier and continuous wavelet transforms, which showed excellent performance on multi-channel signal inputs. Liang P et al. [10] combined CNNs with the wavelet transform and multi-label classification to improve fault diagnosis. He C et al. [11] proposed a novel transmission fault diagnosis approach using MSCNN-LSTM-CBAM-SE, where the outputs of the CBAM-SE module are effectively integrated with the multi-scale features from MSCNNs and the temporal characteristics captured by LSTMs, creating a more comprehensive feature set for diagnosis. Qiao M et al. [12] introduced a dual-input model that integrates CNNs and LSTMs, leveraging both time- and frequency-domain features for end-to-end fault detection. This model employs 1D convolutional and pooling layers to extract the spatial features while retaining the sequential information of the data. Furthermore, Xie et al. [13] applied the Vision Transformer architecture to rolling bearing fault diagnosis. Their method first processes vibration signals using singular value decomposition, then transforms the decomposed signals into two-dimensional image representations. By leveraging the global self-attention mechanism of the Vision Transformer, their approach achieves excellent diagnostic performance, demonstrating the model’s strong capability in capturing comprehensive fault characteristics. S Kumar et al. [14] innovatively proposed a higher-order moment (HOM)-based method for gearbox fault diagnosis. Unlike traditional time-domain features, this approach extracts logarithmic higher-order moment (LHOM) features from the signal amplitudes and combines them with multiple classifiers for fault identification. The results demonstrate that LHOM features can effectively serve as discriminative features for transmission fault diagnosis.

Empirical Mode Decomposition (EMD) is an adaptive signal processing technique that can break down a complex, nonlinear, nonsmooth signal into a set of intrinsic modal functions (IMFs) [15,16]. These IMFs help to capture the local characteristics and time-varying information of the signal, enabling effective noise reduction and interference removal. This results in cleaner and more representative data, which can be used as inputs for subsequent deep learning models. Hu Niaoqing et al. [17] proposed a fault diagnosis method that combines EMD with deep convolutional neural networks (DCNNs). Ali J B et al. [18] effectively achieved bearing fault detection without manual intervention by combining Empirical Mode Decomposition (EMD) with Artificial Neural Networks (ANNs).

Although the above methods have achieved remarkable results in fault diagnosis, they still have some limitations. First, these methods usually rely on complex network structures such as long short-term memory networks (LSTMs) [19], which are capable of extracting rich features, but also bring about high computational complexity and memory consumption, which limits their application in resource-constrained environments. Secondly, these methods often require a large amount of training data to ensure the generalization ability of the model, and in real industrial scenarios, it may be more difficult to obtain a large amount of labeled data. Foreign researchers Woo S et al. [20] proposed the Convolutional Block Attention Module, which can be integrated with any feedforward convolutional neural network, optimizing the classification and detection performance across various models. In addition, Wang et al. [21] proposed a CBAM mechanism, which achieved better results in gearbox fault diagnosis. Although attention mechanisms such as the CBAM [22] can improve the accuracy of feature extraction, their design is usually more complex, which increases the computational burden of the model. Emerging methods such as Transformers [23], although they perform well in dealing with sequential data, may not be sufficiently fine in dealing with local features, and the computational complexity of the multi-attention mechanism is relatively large.

To tackle these challenges, this paper proposes to combine the local feature capture of a multi-scale convolutional neural network (MSCNN) under EMD [24,25], with the local–global feature co-modeling advantage of the lightweight convolutional attention (LCA) mechanism [26,27], and to replace the traditional multiple attention mechanism with the dynamic convolutional kernel generation strategy of LCA [28], and the model can adaptively capture the local features in the time domain and reduce the computational complexity [29,30]. Meanwhile, the exponentially decaying dynamic learning rate and the parametrically revised linear unit (PReLU) [31] are used to achieve fast convergence and preserve fault-sensitive negative information during training. The method can better preserve the fault features in the data processing and reduce the computational effort while achieving fast convergence during training to ensure the accuracy and generalization ability of the model.

## 2. Relevant Theories

### 2.1. Empirical Mode Decomposition

Empirical Mode Decomposition (EMD) is an adaptive signal processing technique primarily used to decompose complex nonlinear and nonsmooth signals into a set of intrinsic mode functions (IMFs) with varying characteristic scales, along with a residual component. The processing steps are as follows.

The original signal is x(t); find out all of the maximum and minimum points of the signal x(t), and connect all of the maximum and minimum points with the cubic spline interpolation method to obtain the upper envelope $e_{max}(t)$ and the lower envelope $e_{min}(t)$.

The local mean m(t) is calculated as shown in Equation (1).

(1) $$m\left(t\right)=\frac{e_{max}\left(t\right)+e_{min}\left(t\right)}{2}$$

Subtract the local mean m(t) from the original signal x(t) to obtain the new signal h(t). Determine whether h(t) satisfies the IMF conditions: in the entire data sequence, the number of extreme points (extreme values and extreme minima) differs from the number of points over zero by, at most, one, and satisfies m(t)≈0. If h(t) satisfies the two conditions of an IMF, then h(t) is recognized as an IMF and denoted as $c_{1}(t)$. If the conditions are not met, the above steps are repeated iteratively until a signal that satisfies the IMF criteria is achieved.

After obtaining the first IMF, the first IMF $IMFc_{1}(t)$ is subtracted from the original signal x(t) to obtain the new remaining signal $r_{1}(t)$ as shown in Equation (2).

(2) $$r_{1}\left(t\right)=x\left(t\right)-c_{1}\left(t\right)$$

Use $r_{1}(t)$ as the new original signal, and repeat the above steps to obtain all IMFs in turn until the remaining signal $r_{n}(t)$ becomes a monotonic function, or the number of its extreme points does not satisfy the conditions for continued decomposition. The final original signal x(t) can be expressed as follows:

(3) $$x\left(t\right)=\sum_{i=1}^{n}c_{i}\left(t\right)+r_{n}\left(t\right)$$

### 2.2. Improved Multi-Scale Convolutional Neural Networks

**Figure 1:**
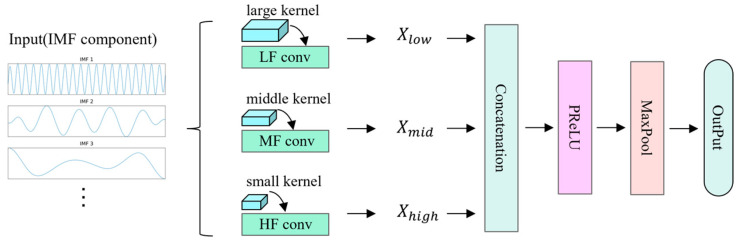
Multi-scale convolutional neural network.

The traditional single-scale convolutional neural network (1D CNN) [32], when dealing with IMF components after EMD, often can only extract the features from a single frequency interval, and it is difficult to comprehensively capture the multi-frequency band characteristics of the gearbox faults by sliding the convolutional kernel in the convolutional layer over the vibration signal data for convolutional operation. For this reason, this study employs a multi-scale convolutional neural network that utilizes three different kernel sizes—large (kernel size = 7), medium (kernel size = 5), and small (kernel size = 3)—to extract the IMF components across low (0–300 Hz)-, medium (300–1000 Hz)-, and high (above 1000 Hz)-frequency bands, respectively. The large kernel benefits from a wider receptive field, enabling it to cover longer signal time spans, and thus better capture the low-frequency components. Conversely, the small kernel’s narrower receptive field exhibits greater sensitivity to transient signal variations, making it particularly suitable for detecting subtle fault characteristics in high-frequency IMF components. These extracted features are then integrated through a parallel multi-branch architecture to comprehensively capture the multi-band characteristics of the IMF components, as illustrated in Figure 1. This approach significantly enhances the model’s adaptability to complex operational conditions.

After the convolution operation, a parameterized corrected linear unit is used for nonlinear activation, and the PReLU function handles the positive and negative inputs differently: for positive inputs, the output is a linear value; for negative inputs, the output is adjusted according to the learnable parameter α. When dealing with fault data, the network can learn to optimize the value of α so that the output can be performed in a more appropriate way when dealing with negative features associated with faults, as shown in Equation (4).

(4) $$f\left(x\right)=\left\{\begin{array}{c} x\;,\;\;\;\;\;\;\;x\geq 0\\ \alpha x\;,\;\;\;\;x<0 \end{array}\right.$$

Then enters the maximum pooling layer, which reduces the data dimensions, reducing the amount of data to be processed for subsequent calculations while also carrying out the further extraction of the main features.

### 2.3. LCAEncoder

The gearbox vibration signal presents multi-scale time–frequency characteristics after EMD, and the traditional self-attention mechanism [33] or multi-head attention mechanism [34] suffers from the high computational complexity and blurred local features. In contrast, lightweight convolutional attention (LCA) significantly improves the computational efficiency and local feature capture capability, while maintaining the global modeling capability by introducing dynamic convolutional kernel generation [35] and multi-head group convolution, and the core formulation of the LCA is as follows:

(5) $$LCA\left(X\right)=DepthwiseConv1D\left(X,W_{kernel}\right)+X$$

(6) $$W_{kernel}=Softmax\left(W_{g}\cdot GAP(X)\right)+X$$

where $W_{kernel}$ is the dynamically generated convolutional kernel weight, H is the number of attention heads, and K is the convolutional kernel size; $W_{g}$ is the learnable parameter matrix, and GAP(X) is the global averaging effect of input X.

In addition, LCA explicitly models the local differential features through the local receptive fields of the convolutional kernel, which can capture the shock characteristics in gear faults more efficiently:

(7) $$\frac{\partial f(t)}{\partial t}\approx \sum_{k=-\frac{K}{2}}^{\frac{K}{2}}w_{k}.f(t+k)$$

This local feature enhancement mechanism makes LCA more sensitive to early faults, such as pitting and cracking of the gears. Meanwhile, the dynamic convolutional kernel weights $W_{kernel}$ of the LCA are generated by global feature adaption, which overcomes the limitation of the static weights of the traditional convolutional kernels and can better adapt to non-stationary vibration signals under variable speed conditions.

**Figure 2:**
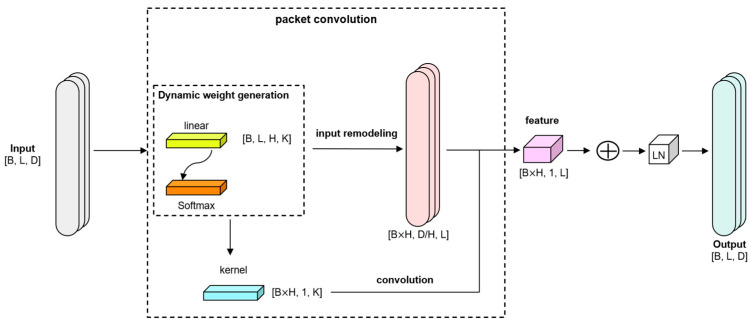
The LCA module generates dynamic convolutional kernels and performs grouped convolution operations.

The principle of LCA is shown in Figure 2. Firstly, the dynamic convolutional kernel is automatically generated from the input; then, the input is reshaped and split into H-heads, and then grouped into H-groups for group convolution. Then, the output is projected, and finally, residual joining and layer normalization are performed.

In LCAEncoder, the authors use LCA to replace the multi-head attention mechanism in the traditional Transformer, but retain the linear layer, Norm layer, and dropout layer in the Transformer. The complexity and advantages are compared in Table 1:

### 2.4. Dynamic Learning Rate

In the case of gearboxes, where the signals of the fault characteristics are not too obvious, using a fixed learning rate can create a trade-off between fast convergence during the initial phase and achieving an accurate convergence in the later stages. Setting the learning rate too high risks destabilizing the convergence during the later stages, whereas an insufficiently low rate impedes efficient optimization in the early training period. Therefore, this paper proposes to use a dynamic learning rate to solve this problem.

**Figure 3:**
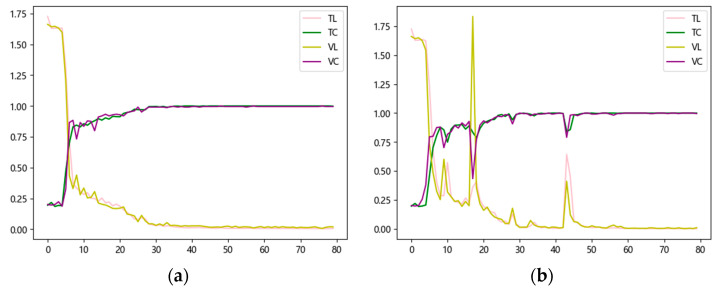
Learning rate strategy comparison. (**a**) Exponential decay dynamic learning rate; (**b**) fixed learning rate.

At the same time, the change of the learning rate should match the training process. To this end, this paper experimentally tests three common dynamic learning rate methods: the cosine annealing method, the exponential decay method, and the segmentation constant method. The dynamic learning rate strategy is globally applied to all trainable parameters, encompassing both the multi-scale convolutional module and the LCA module. The experimental findings reveal that the cosine annealing method reduces the learning rate too quickly in the later stages, making it challenging for the model to converge stably; the segmented constant method lacks flexibility and is prone to missing the optimal solution. Finally, the experimental results demonstrate that the exponential decay method is the most suitable for meeting the training requirements of the model in this study. The comparative results between the exponential decay dynamic learning rate and the fixed learning rate during the training process are illustrated in Figure 3. The curves in the figure, respectively, demonstrate the training set accuracy (TC) and training set loss (TL), as well as the validation set accuracy (VC) and validation set loss (VL). The principle of the exponential decay method is shown in the following equation.

(8) $$lr_{t}=lr_{0}\times \gamma^{t}$$

where $lr_{0}$ denotes the initial learning rate, y denotes the decay factor, t denotes the number of training rounds, and $lr_{t}$ denotes the learning rate at the tth round of training. The results demonstrate that the exponential decay dynamic learning rate strategy outperforms the fixed learning rate approach in both convergence speed and accuracy stability.

The initial learning rate should be set according to the actual demand, while the smaller the attenuation factor is, the faster the final learning rate decreases. Therefore, the selection of the decay factor also needs to be adjusted according to the final convergence accuracy requirements.

## 3. Proposed Method

**Figure 4:**
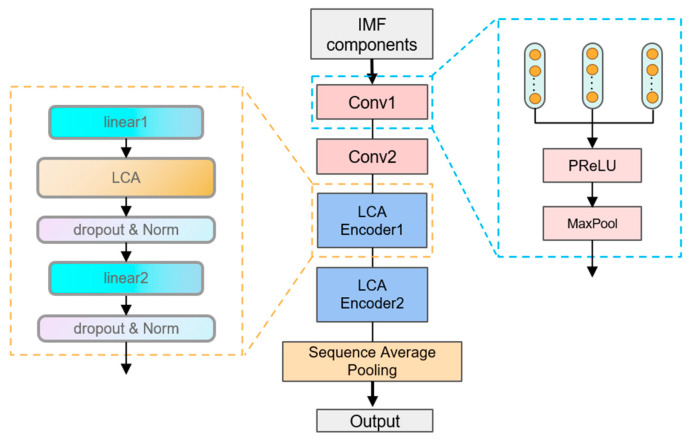
Structure of MSCNN-LCA-Transformer.

This study first employs Empirical Mode Decomposition to preprocess the segmented raw vibration signals. Through adaptive decomposition, seven IMF components are obtained as the foundation for the subsequent analysis. These IMF components completely preserve the time–frequency characteristics of the original signals. The preprocessed data are then sequentially fed into a two-layer MSCNN, followed by a two-layer LCAEncoder. The model is able to efficiently extract the fault features through multi-scale convolution, pooling, and the LCA mechanism. Next, the features are further processed using a sequence-averaged pooling operation. The structure of the model is shown in Figure 4, which not only can effectively retain the fault features and reduce the data dimensionality, but also can adapt to different input lengths, providing a solid foundation for the subsequent classification and prediction tasks.

In the LCAEncoder, two linear, dropout, and normalization layers each are set up. The linear layer performs a linear transformation on the input data and adjusts the feature representation of the input data to make them more suitable for subsequent processing. The dropout layer randomly sets the output of a portion of neurons to 0 during the training process, which prevents model overfitting. The normalization layer normalizes for the specific dimensions of each sample, which helps to stabilize the training process of the model and improves the convergence speed and generalization ability of the model.

## 4. Experimental Verification

### 4.1. Troubleshooting Open-Source Datasets

**Figure 5:**
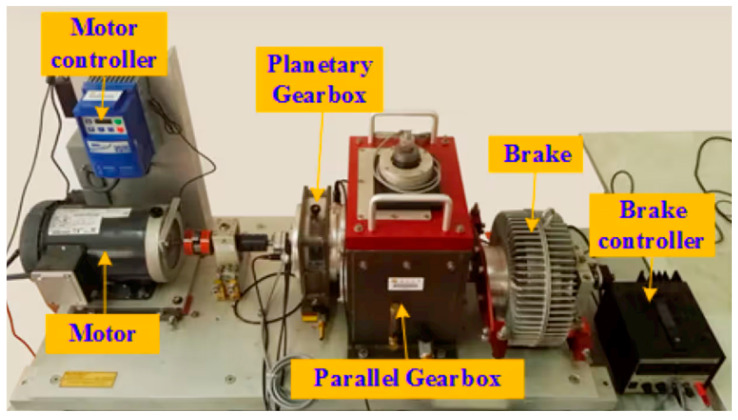
Driveline simulator.

To validate the model’s performance, the gearbox dataset from Southeast University was utilized in the experiments. This dataset is derived from the Driveline System Simulator, and the experimental equipment is shown in Figure 5.

**Figure 6:**
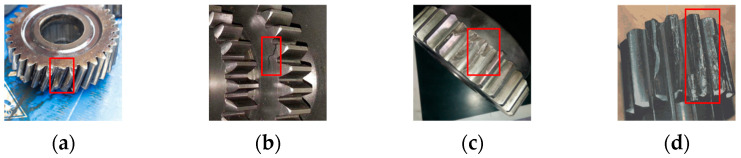
Examples of failures of different gears, with fault characteristics highlighted in red boxes. (**a**) Broken tooth; (**b**) root crack; (**c**) missing teeth; (**d**) tooth wear.

This dataset contains vibration signals from the parallel and planetary gearboxes in the x, y, and z axes, along with motor vibration signals from the z axis and torque data. Normal conditions and four gear failure conditions, namely broken teeth, missing teeth, root cracks, and tooth wear, were simulated, as shown in Figure 6. The datasets of two working conditions were used in the experiment. Working condition 1: the gearbox speed was set to 1200 rpm, the load to 0 Nm, and the sampling frequency to 5120 Hz. Working condition 2: the gearbox speed was set to 1800 rpm, the load to 7.32 Nm, and the sampling frequency to 5120 Hz.

The gearbox fault diagnosis based on the MSCNN-LCA-Transformer was divided into five parts: signal processing division, signal EMD, creation of the dataset, MSCNN-LCA-Transformer model training, and fault diagnosis.

**Figure 7:**
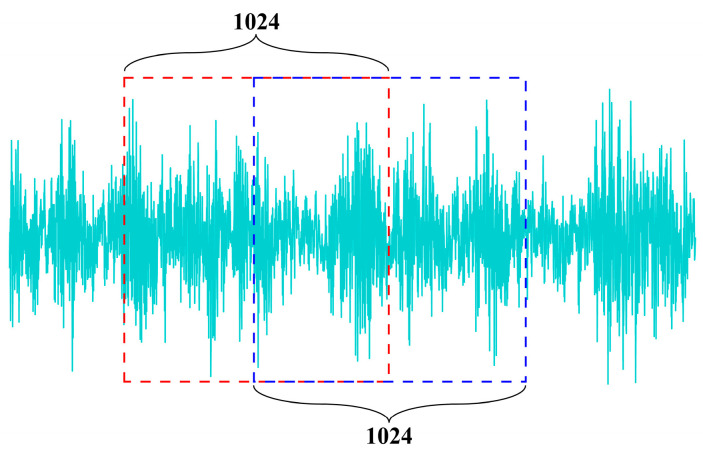
Signal segmentation example. (The red box represents the first 1024 points, the blue box indicates the last 1024 points, with a 50% overlap).

**Figure 8:**
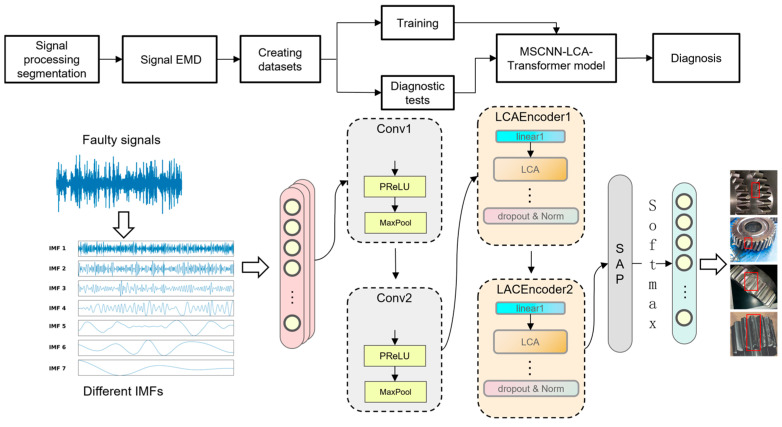
Diagnostic process.

First, as illustrated in Figure 7, the dataset was segmented into samples with a fixed time-step length of 1024 points, employing a 50% overlap rate between adjacent segments. Subsequently, the dataset was partitioned into training, validation, and test sets at ratios of 70%, 20%, and 10%, respectively. Following this division, each sample underwent Empirical Mode Decomposition, with the first seven IMF components retained for further analysis. The final processed dataset is summarized in Table 2. The complete process is shown in Figure 8.

After repeated experiments to adjust the relevant parameters, the final model parameters obtained are shown in Table 3. IC denotes the number of input channels of the convolutional layer; OC denotes the number of output channels of the convolutional layer; k denotes the size of the convolutional kernel; stride denotes the moving step of the convolutional kernel; padding denotes the number of padding at the edge of the data; AT denotes the input feature dimensions; H denotes the number of heads of attention, which is also the number of random groupings; K denotes the time-step span of the convolutional kernel; and dropout denotes that it randomly sets the neuron’s output to 0 according to a certain probability during the training process, which can prevent the model from being overfitted to a certain extent and at the same time enhance the model’s generalization ability.

**Figure 9:**
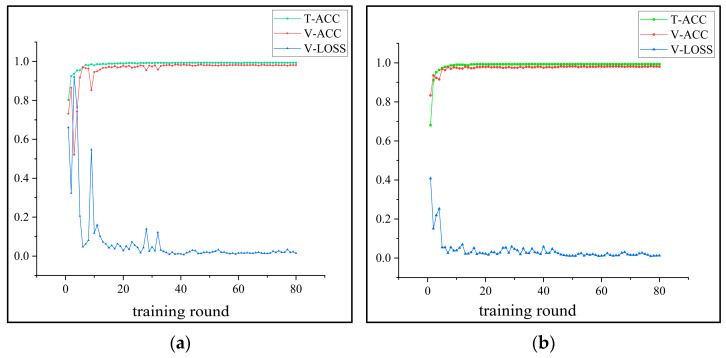
The training accuracy curves for the Southeast University gearbox dataset. (**a**) Condition 1; (**b**) condition 2.

**Figure 10:**
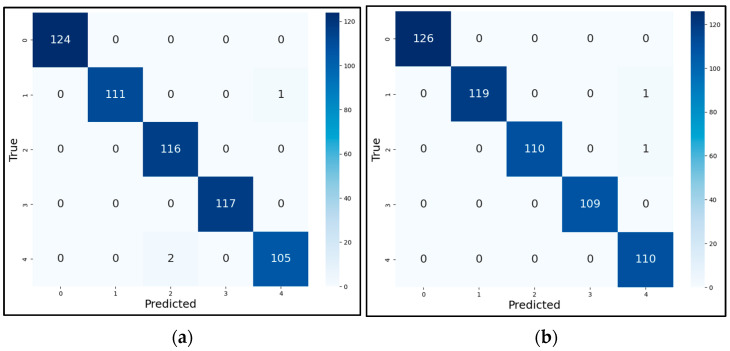
Confusion matrices of test set diagnostic results. (**a**) Condition 1; (**b**) condition 2.

The model employed the AdamW optimizer to adapt the learning rate for each parameter; each round of training was verified by the validation set, and the model was updated according to the validation results. The final convergence curves of the training set accuracy (T-Acc), validation set accuracy (V-Acc), validation set loss (V-Loss), and the resultant confusion matrices of the test set of the MSCNN-LCA-Transformer model optimized using the PReLU activation function and the exponentially decaying dynamic learning rate under two working conditions are shown in Figure 9 and Figure 10.

**Figure 11:**
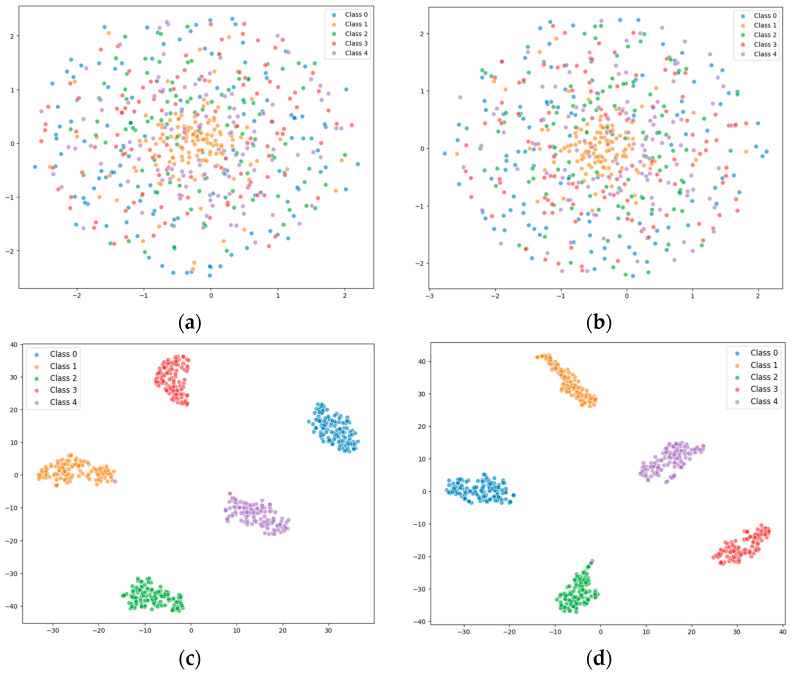
Feature distribution visualization through t-SNE. (**a**) Raw data for condition 1; (**b**) raw data for condition 2; (**c**) data after classification for condition 1; (**d**) data after classification for condition 2.

The evaluation metrics, such as precision and recall, after running the model are shown in Table 4. The feature distributions of both the raw data and the classified data were visualized using t-SNE in our experiments, as shown in Figure 11. It can be seen that the MSCNN-LCA-Transformer model converges quickly, and the final diagnostic precision for health and the four kinds of faults reaches 99.4%, which can achieve excellent diagnostic results. This performance validates the effectiveness of the LCA module in local feature enhancement, and the hybrid architecture combining multi-scale convolution and lightweight convolutional attention mechanisms successfully achieves the collaborative extraction of multi-scale time–frequency features.

### 4.2. Generalizability Experiments

To validate the generalization capability of the proposed model, we evaluated its fault diagnosis performance on the WT planetary gearbox dataset using the aforementioned model architecture and signal processing methods. The experimental dataset, characterized by a sampling frequency of 48 kHz and a rotational speed of 40 Hz (2400 rpm), includes vibration data from both the x and y axes. Compared to the Southeast University dataset, this test scenario involves higher rotational speeds and stronger noise interference. The experimental results are presented below.

**Figure 12:**
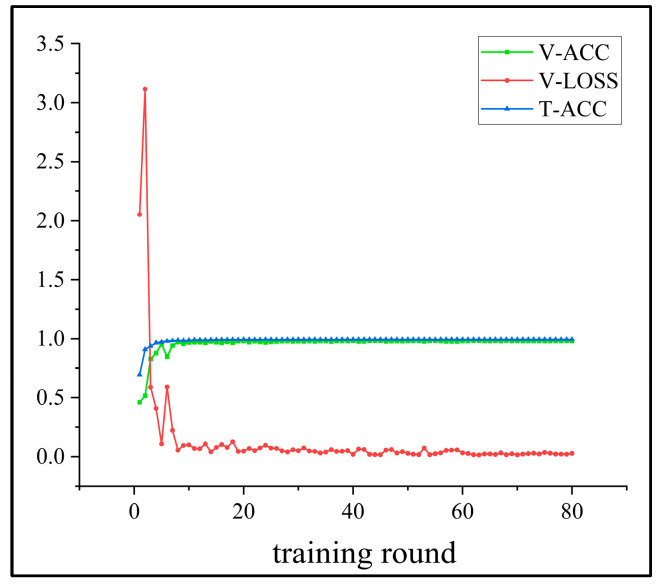
The training accuracy curves for the WT planetary gearbox dataset.

**Figure 13:**
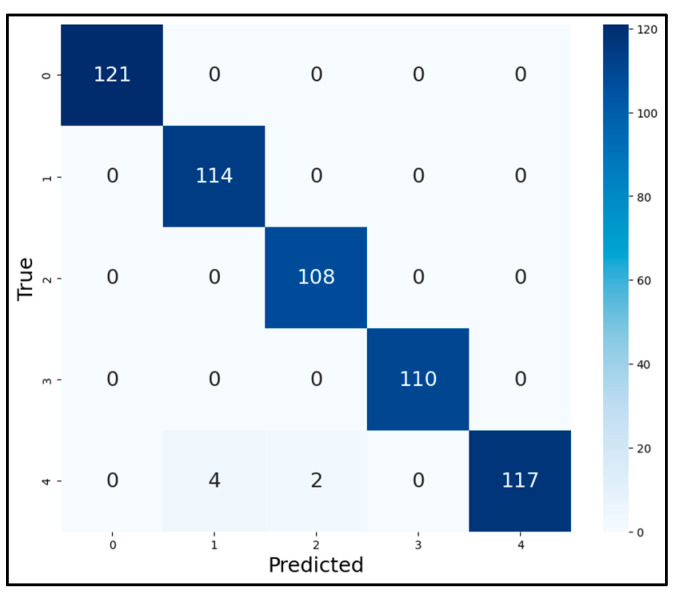
Confusion matrix of test set diagnostic results.

**Figure 14:**
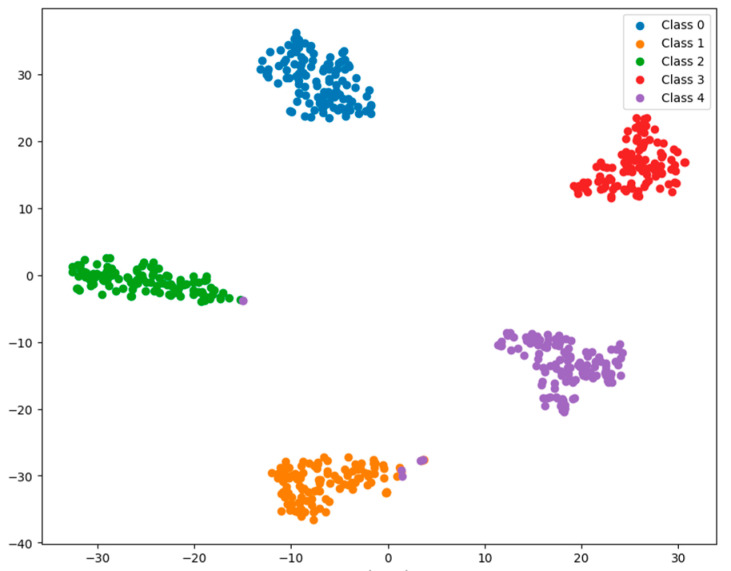
Feature distribution visualization through t-SNE.

As shown in Figure 12, although the validation loss is relatively high during the first two training epochs, the model demonstrates rapid convergence after the seventh epoch while maintaining stable accuracy. The final test set accuracy reaches 98.9%, with the corresponding confusion matrix and feature distribution visualizations presented in Figure 13 and Figure 14, respectively. The quantitative evaluation metrics, including precision and recall rates, are summarized in Table 5.

Based on the experimental results presented above, the proposed model demonstrates robust performance across diverse datasets, confirming its strong generalization capability.

### 4.3. Comparison Tests

To further validate the performance of the proposed model, we conducted comparative experiments using the Southeast University gearbox dataset under operational condition 2 (rotational speed: 1800 rpm; load: 7.32 N·m). This operating condition, characterized by stronger noise interference and more subtle fault signatures, serves as an effective testbed for evaluating the model’s robustness.

Four comparison models—a CNN-LCA-Transformer, a MSCNN-CBAM-Transformer, a MSCNN-Transformer, and a MSCNN-BiLSTM—were set up in the experiments to verify the superiority of the MSCNN-LCA-Transformer model proposed in this paper. The number of training rounds for each experiment was set to 80, and the diagnostic results of the test set were taken as the average of eight experiments.

**Figure 15:**
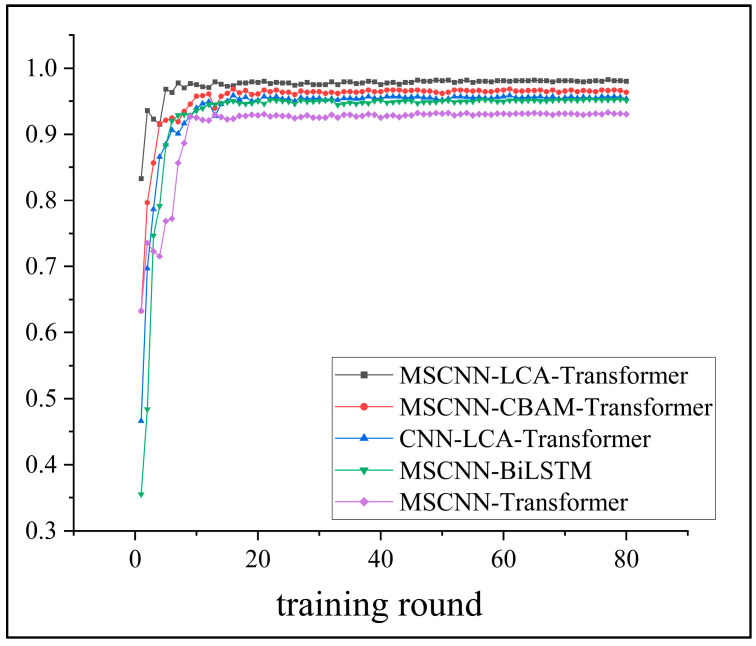
Comparison of accuracy of different models.

The accuracy curves of the different models on the validation set during training are shown in Figure 15, and the average accuracy of each model on the diagnostic test set is listed in Table 6. From the results, it can be seen that the MSCNN and LCA have improved performance compared to the traditional CNNs and Transformers, and the MSCNN-LCA-Transformer model proposed in this paper is also the best in terms of both convergence speed and diagnostic accuracy compared to the other models.

## 5. Conclusions

In this study, an innovative gearbox fault diagnosis method based on a multi-scale convolutional neural network and a lightweight convolutional attention mechanism is proposed. The core innovation lies in the combination of the MSCNN and LCA mechanisms, which achieves the accurate extraction of the multi-level features of vibration signals and effectively improves the modeling capability of the model on global features by dynamic convolutional kernel generation and multi-head group convolution, while reducing the computational complexity.

The experimental data indicate that the proposed method performs exceptionally well in gearbox fault diagnosis, particularly in terms of diagnostic accuracy and convergence speed, which are significantly better than those of traditional methods. The diagnostic accuracy reaches more than 98.9% on both variable operating condition datasets. Compared with other existing fault diagnosis methods, the proposed method shows obvious advantages in several performance indicators and has stronger robustness and generalization ability. This method provides a new solution for gearbox fault diagnosis, which is of great significance for promoting the development of intelligent fault diagnosis technology. Future research will further optimize the model architecture and extend it to other mechanical equipment fault diagnosis tasks to verify its universality and practicality.

## Figures and Tables

**Table 1:** Complexity and advantages in comparative analysis.

**Indicators**	Multi-Head Self-Attention	LCA
Computational complexity	$O(L^{2}D)$	O(LKD)(K≪L)
Number of parameters	$O(DH^{2})$	O(HDK)
Dynamic adaptation	Not have	Dynamic generation of convolutional kernel parameters
Noise immunity	Easy diffusion of noise	Local convolution suppresses noise propagation

**Table 2:** Dataset description.

Gear State	Training Set	Validation Set	Test Set	Category Labeling
Wellness	815	233	117	1
Broken teeth	815	233	117	2
Missing teeth	815	233	117	3
Root crack	815	233	117	4
Surface wear	815	233	117	5

**Table 3:** The main parameters of the model.

Structures	Parameters
Conv1	IC = 56; OC = 64; k = 7\5\3; stride = 1; padding = 3\2\1
MaxPool1	k = 2; stride = 2
Conv2	IC = 64; OC = 128; k = 7\5\3; stride = 1; padding = 3\2\1
MaxPool2	k = 2; stride = 2; padding = 1
Encoder1	AT = 128; H = 4; K = 15
Encoder2	AT = 128; H = 4; K = 15
Dropout	Discard rate: 0.2
Dynamic learning rate	Initial learning rate: 0.001; Attenuation factor: 0.95

**Table 4:** Evaluation metrics.

Categorization	Precision	Recall	F1-Score
0	1	1	1
1	1	0.992	0.996
2	1	0.991	0.996
3	1	1	1
4	0.982	1	0.991

**Table 5:** Evaluation metrics.

Categorization	Precision	Recall	F1-Score
0	1	1	1
1	0.9661	1	0.9828
2	0.9818	1	0.9908
3	1	1	1
4	1	0.9512	0.975

**Table 6:** Accuracy of different model test sets.

Model	Test Set Accuracy %	Training Time (s)
MSCNN-LCA-Transformer	99.46	687
MSCNN-CBAM-Transformer	97.95	890
CNN-LCA-Transformer	96.69	693
MSCNN-BiLSTM	96.26	824
MSCNN-Transformer	94.29	893

## Data Availability

The open-source Southeast University gearbox dataset used in the experiment, including both operating condition 1 and operating condition 2, can be accessed through the following link: https://github.com/cathysiyu/Mechanical-datasets/tree/master/gearbox/gearset (accessed on 17 December 2024) The WT planetary gearbox open-source dataset is available at the following link: https://github.com/Liudd-BJUT/WT-planetary-gearbox-dataset (accessed on 8 April 2025).

## Acronym List

EMD—Empirical Mode Decomposition; LCA—Lightweight Convolutional Attention; MSCNN—Multi-Scale Convolutional Neural Network; PReLU—Parameterized Rectified Linear Unit; IMF—Intrinsic Mode Function; LSTM—Long Short-Term Memory; BiLSTM—Bidirectional Long Short-Term Memory; CBAM—Convolutional Block Attention Module.
